# Reirradiation for Patients With Recurrent Ependymoma Across the Age Spectrum

**DOI:** 10.1016/j.adro.2026.102039

**Published:** 2026-04-02

**Authors:** Ying Ying Sum, Julie Bennett, Vijay Ramaswamy, Anirban Das, Anthony P. Liu, Annie Huang, Uri Tabori, Peter Dirks, Farshad Nassiri, Dana M. Keilty, Derek S. Tsang

**Affiliations:** a Radiation Medicine Program, Princess Margaret Cancer Centre, University Health Network, Toronto, Canada; b Division of Haematology/Oncology, Hospital for Sick Children, Toronto, Canada; c Division of Medical Oncology and Hematology, Princess Margaret Cancer Centre, Toronto, Canada; d Division of Neurosurgery, Hospital for Sick Children, Toronto, Canada; e Division of Neurosurgery, University Health Network, Toronto, Canada

## Abstract

**Purpose:**

In patients with locally recurrent ependymoma, previous data have shown that repeat radiation therapy (RT2) with craniospinal irradiation (CSI) improves disease control compared with focal RT2 alone. This study aimed to report long-term outcomes of adults and children with recurrent intracranial ependymoma, including those treated with CSI.

**Methods and Materials:**

In this retrospective, single-institution study, we identified 48 patients treated with RT2 for recurrent ependymoma between 1999 and 2024. The primary endpoint was overall survival (OS) with the start of RT2 as the index time. CSI was prescribed to 23.4 or 36 Gy, followed by focal boost radiation therapy to 54 Gy. OS was estimated using the Kaplan-Meier method; groups were compared using the log-rank test.

**Results:**

The median age at initial diagnosis was 6.5 years (range, 0.8-62.3 years), and the majority of patients were pediatric (83%; aged <18 years) with a posterior fossa (PF) tumor location (67%). Of the 35 patients with available molecular pathology, 23 had PF-A, and 11 had a zinc finger translocation associated fusion. The median follow-up was 9.1 years. Gross total resection of all disease prior to RT2 was associated with better 5-year OS (57% vs 19%; *P* = .015). Among the 33 patients (69%) with only local recurrence, the 5-year OS was significantly higher with CSI-RT2 (n = 10) than with focal RT2 (n = 23) (78% vs 36%, respectively; *P* = .040). In the subgroup of 24 PF local recurrences, the 5-year OS was 83% with CSI (n = 7) and 30% with focal RT2 (n = 17) (*P* = .061). The 5-year OS for the 15 patients with disseminated-only or synchronous local and disseminated failures was 20%; all but 1 were treated with CSI-RT2.

**Conclusions:**

In this single-institution cohort of patients with recurrent ependymoma and long-term follow-up, gross total resection of recurrence was associated with better survival, and locally recurrent patients treated with CSI-RT2 had longer OS. Further work is required to determine the independent effects of CSI-RT2 together with the extent of re-resection.

## Introduction

Ependymoma is a rare primary central nervous system tumor arising from ependymal cells lining the ventricular system and spinal canal. It accounts for approximately 10% of pediatric intracranial tumors and a smaller proportion of adults.^1^ Standard management includes maximal safe surgical resection, which remains the cornerstone of therapy, followed by adjuvant radiation therapy (RT).^2^

Recurrence remains relatively common despite aggressive multimodal treatment; in a multi-institutional, prospective Children’s Oncology Group study,^3^ 5-year progression-free survival (PFS) was only 63%. Recurrent ependymoma poses a significant therapeutic challenge due to frequent local and sometimes disseminated relapse in the background of prior treatment-related constraints and previous radiation dosing. Surgical resection at recurrence is associated with improved survival,^4^ but complete resection is often difficult due to anatomic limitations.^5^

Reirradiation (RT2) has gained increasing support as a salvage strategy, with several studies reporting its feasibility and clinical benefit.^6^, ^7^, ^8^ RT2 to the pediatric brain and brainstem at cumulative doses higher than what would typically be regarded as normal tissue tolerance has been found to be well tolerated, with minimal rates of radiation necrosis.^9^ Notably, a previously published study from our institution demonstrated that craniospinal irradiation (CSI) in pediatric patients with localized recurrence resulted in improved disease control and survival compared with focal RT2.^10^

This study is an update of our institutional data, with longer follow-up and a larger cohort, including adult patients, with the goal of critically evaluating the roles of repeat surgical resection and CSI in patients with recurrent intracranial ependymoma.

## Methods and Materials

This retrospective cohort study was conducted at a single institution. We identified all patients diagnosed with intracranial ependymoma who underwent RT2 for recurrent disease. Eligible patients had a pathologically confirmed diagnosis of ependymoma and received focal initial RT (RT1) for localized disease at initial diagnosis between 1999 and 2024, followed by subsequent relapse and receipt of a second course of radiation.

Patterns of recurrence were defined radiographically relative to the initial RT1 field. Patients with relapse within the original tumor bed were considered to have local recurrence. Patients with relapse distant to the site of the original disease were considered to have disseminated recurrence. Those with synchronous local and disseminated failures were analyzed within the disseminated group.

For infratentorial tumors, molecular subgrouping was performed on selected specimens obtained at the time of initial diagnosis using genome-wide DNA methylation profiling or H3K27me3 immunohistochemistry. Posterior fossa (PF) tumors were classified into group A (PF-A) and group B (PF-B). In supratentorial tumors, zinc finger translocation associated gene fusion was documented when available.

Patients underwent repeat surgery at recurrence, when possible. The extent of resection prior to RT2 was determined from postoperative imaging and operative reports and categorized as gross total resection (GTR), near-total resection, subtotal resection (STR), or biopsy only. Radiation necrosis was graded according to the National Cancer Institute Common Terminology Criteria for Adverse Events, version 5.0.

### RT

RT procedures have been previously described.^10^ RT1 consisted of 59.4 Gy in 1.8 Gy daily fractions to the postoperative tumor bed. For patients who relapsed with localized disease, our institutional practice was to offer focal RT2 until 2011. From 2012 to 2024, CSI, as a component of RT2 (CSI-RT2), was adopted as a standard option for patients with isolated focal relapse for all tumors, regardless of initial location. After CSI-RT2, sequential focal RT2 boosts were delivered to the recurrent tumor bed. From 2019 onward, patients with locally recurrent ependymoma and an initial PF location were offered CSI-RT2 plus a focal RT2 boost, while patients with locally recurrent ependymoma and an initial supratentorial tumor location were treated with focal RT2 alone. CSI-RT2 doses ranged from 23.4 to 36 Gy (at the treating oncologist’s discretion, with a lower dose offered to younger patients) for locally recurrent disease and 36 Gy for disseminated recurrence, all delivered in 1.8 Gy daily fractions.

All patients were treated with photon external beam RT2 at recurrence. At RT2, brainstem, spinal cord, optic chiasm, and optic nerve doses were limited to a maximum of 54 Gy, aiming for ≥95% planning target volume coverage with ≥95% of the RT2 prescription dose; no cumulative dose limits were applied.

### Statistical analysis

Clinical characteristics were reported descriptively. The primary endpoint was overall survival (OS), defined as the time from the start of RT2 to death. PFS was defined as the time from the first day of RT2 to disease recurrence or death. Survival was estimated using the Kaplan-Meier method, with living patients censored at the last follow-up. Differences between groups were compared using the log-rank test; *P* values < .05 were considered statistically significant. Multivariable regression was not performed due to the small sample size. Statistical analyses were performed using SAS version 9.4 (SAS Institute). This study was approved by the hospital research ethics board.

## Results

A total of 48 patients met eligibility criteria and were included in the analysis. Patient demographics and tumor characteristics are summarized in Table 1. The median age at initial diagnosis was 6.5 years (range, 0.8-62.3 years), with most patients being pediatric (defined as age <18 years; n = 40, 83%) and a male predominance (n = 29, 60%). Most tumors (n = 32, 67%) were located in the PF. Among the 35 patients (73%) for whom molecular pathology was available, 23 (66%) had PF-A tumors, 1 (3%) had a PF-B tumor, and 11 (31%) had zinc finger translocation associated fusion-positive tumors.

**Table 1:** Patient demographics and tumor characteristics

Variable	Category	N = 48, n (%)
Age at diagnosis (y), median (range)	-	6.5 (0.8-62.3)
Sex	Men Women	29 (60) 19 (40)
Pediatric (age <18 y at diagnosis)		40 (83)
Site of original disease	Supratentorial Infratentorial	16 (33) 32 (67)
Initial surgery	GTR NTR STR	24 (50) 12 (25) 12 (25)
Histology	Grade 3 Grade 2	30 (63) 18 (34)
Molecular profile	PF-A PF-B ZFTA fusion Unknown	23 (48) 1 (2) 11 (23) 13 (27)
RT1 total dose, Gy	59.4 55.8 54 50.4	28 (58) 18 (38) 1 (2) 1 (2)
Chemotherapy at initial presentation	Yes No	12 (25) 36 (75)
RT1 pattern of failure	Local Disseminated Combined	33 (69) 10 (21) 5 (10)
Surgery at relapse	GTR NTR STR Biopsy only None	26 (54) 3 (6) 7 (15) 3 (6) 9 (19)
RT2 field	Focal CSI	24 (50) 24 (50)
RT2 total dose, Gy	54 55.8 59.4 30 (10 fractions)* 24 (1 fraction)† 15 (1 fraction)†	36 (75) 1 (2) 8 (17) 1 (2) 1 (2) 1 (2)
CSI-RT2 dose, Gy	Locally recurrent: 36 23.4 Disseminated recurrence: 36 23.4	4 (40) 6 (60) 13 (93) 1 (7)
RT2 technique	3DCRT IMRT VMAT SRS	3 (6) 23 (49) 19 (40) 2 (4)

All radiation was delivered in 1.8 Gy daily fractions unless otherwise specified.

*Abbreviations:* 3DCRT = three-dimensional conformal radiation therapy; CSI = craniospinal irradiation; GTR = gross total resection; IMRT = intensity-modulated radiation therapy; NTR = near-total resection; PF-A = posterior fossa tumor group A; PF-B = posterior fossa tumor group B; RT1 = initial radiation therapy; RT2 = reirradiation; STR = subtotal resection; SRS = stereotactic radiosurgery; VMAT = Volumetric modulated arc therapy; ZFTA = zinc finger translocation associated.

⁎ This patient with disseminated (spinal) recurrence after RT1 was retreated with 30 Gy in 10 fractions to the entire spine.

† Stereotactic radiosurgery.

The median follow-up time for RT2 was 9.1 years. The median time interval from RT1 to RT2 was 2.4 years (range, 0.5-11.7 years). Four patients were treated with adjuvant proton therapy upfront (RT1). All courses of RT2 were delivered with photon radiation. Among patients treated with CSI-RT2, the median ages at RT2 were 6.1 years (range, 4.7-8.4 years) and 11.5 years (range, 5.0-29.8 years) for those receiving 23.4 Gy and 36 Gy, respectively.

In the subgroup of 33 patients with locally recurrent tumors after RT1, 10 were treated with CSI-RT2, while 23 were reirradiated with focal RT2. In the CSI group, 8 had GTR, 1 had STR, and 1 had a biopsy prior to RT2; in the focal RT2 group, 12 had GTR, 1 had near-total resection, 5 had STR, 1 had a biopsy, and 4 had no surgery prior to RT2. The proportion of patients undergoing GTR prior to CSI-RT2 was 80%, compared with 52% in the focal RT2 group (*P* = .25).

### Extent of resection

**Figure 1:**
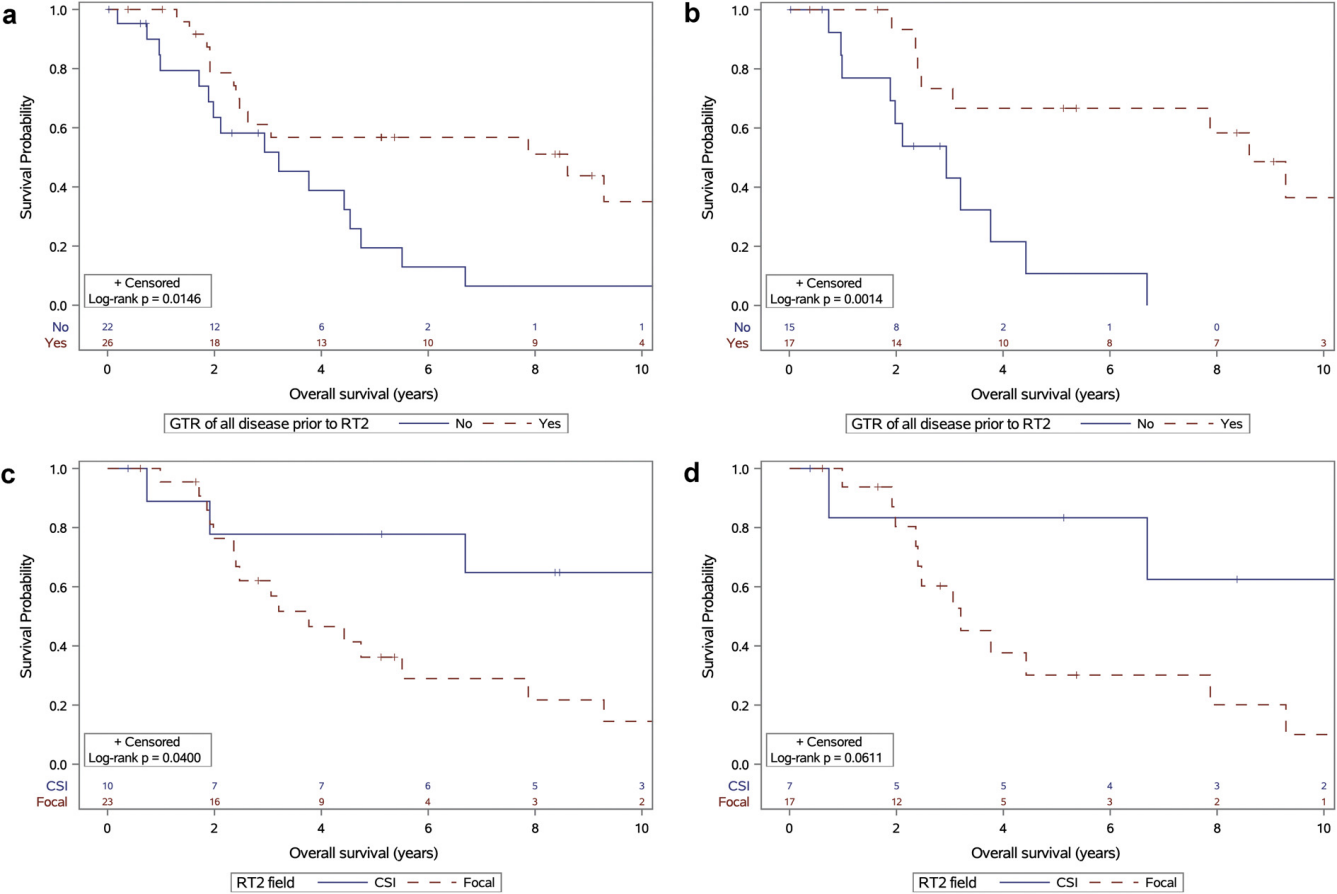
(a) Overall survival by extent of resection at recurrence after initial radiation therapy (gross total resection [GTR] vs not) for all patients. (b) Overall survival by extent of resection in patients after initial radiation therapy with posterior fossa tumors. (c) Overall survival of patients with local recurrence treated with repeat radiation therapy (RT2) with craniospinal irradiation (CSI) versus focal RT2. (d) Overall survival in patients with posterior fossa tumors with local recurrence treated with CSI-RT2 versus focal RT2.

GTR of all recurrent disease prior to RT2 was achieved in 54% of patients and was significantly associated with improved OS. Patients who underwent GTR had a 5-year OS of 57%, compared with 19% in those with STR or biopsy only (*P* = .015; Fig. 1a). This survival difference was also observed in the subgroup of patients with PF tumors, with a 5-year OS of 67% in those who underwent GTR compared with 11% in those who underwent STR or biopsy only (*P* = .001; Fig. 1b).

**Figure 2:**
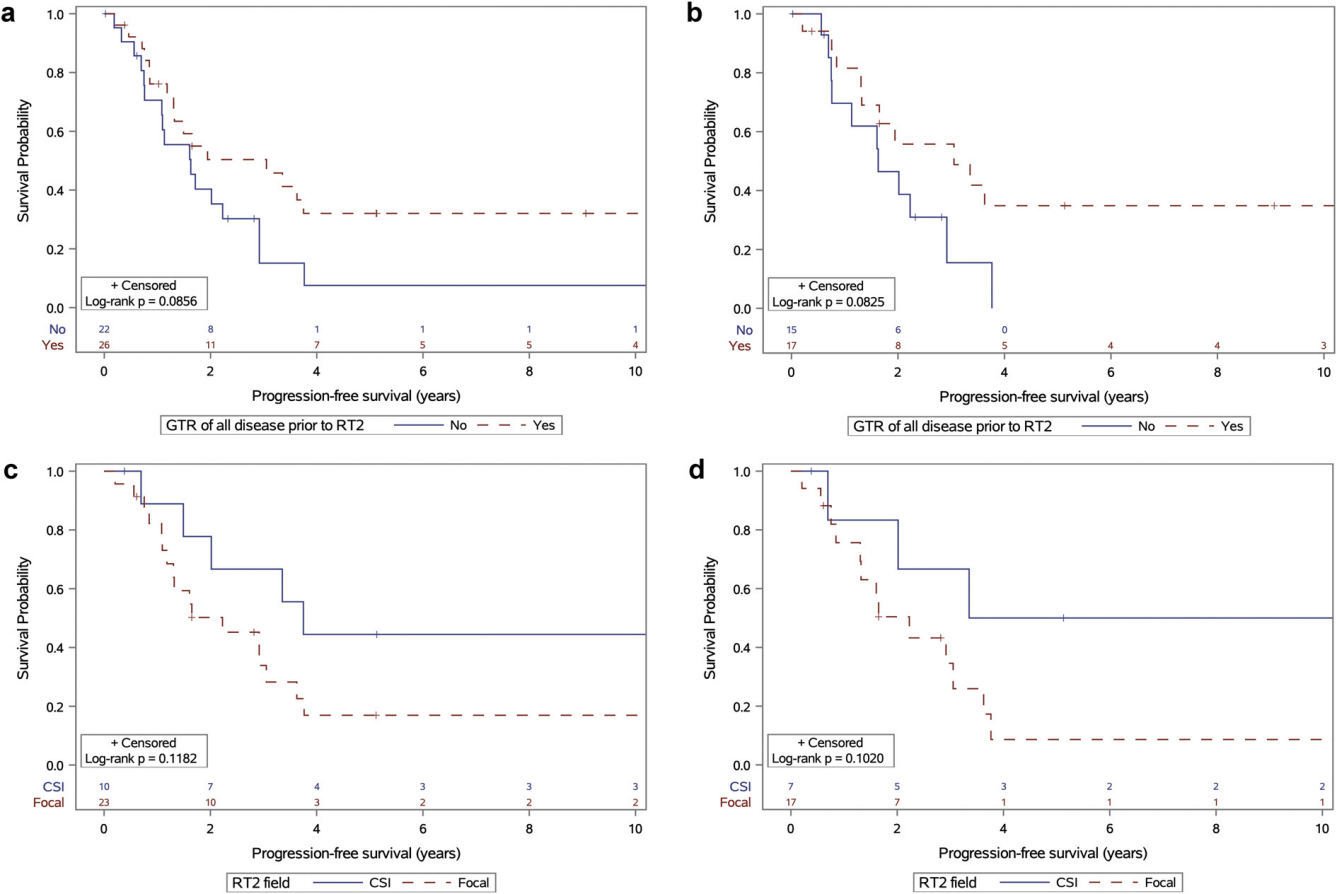
(a) Progression-free survival by extent of resection at recurrence after initial radiation therapy (gross total resection [GTR] vs not) for all patients. (b) Progression-free survival by extent of resection at recurrence after initial radiation therapy in patients with posterior fossa tumors. (c) Progression-free survival of patients with local recurrence treated with repeat radiation therapy (RT2) with craniospinal irradiation (CSI) versus focal RT2. (d) Progression-free survival in patients with posterior fossa tumors with local recurrence treated with CSI-RT2 versus focal RT2.

Similarly, the extent of resection trended toward an association with PFS. Across the entire patient cohort, those who underwent GTR had a 5-year PFS of 32%, compared with 7% in those who did not (*P* = .086; Fig. 2a). In the subgroup of patients with PF tumors, those who underwent GTR had a 5-year PFS of 35% versus 0% with incomplete resection (*P* = .083; Fig. 2b).

### CSI

Among the 33 patients who experienced only local recurrence after RT1, treatment with CSI-RT2 (n = 10) resulted in significantly better 5-year OS than focal RT2 alone (n = 23) (78% vs 36%, respectively; *P* = .040; Fig. 1c). In the subgroup of 24 patients with PF tumors and isolated local recurrence, CSI-RT2 showed a trend toward superior survival compared with focal RT2, with a 5-year OS of 83% versus 30%, respectively, though this did not reach statistical significance (*P* = .061; Fig. 1d).

Among patients with local recurrence after RT1, treatment with CSI-RT2 yielded a 5-year PFS of 44% compared with 17% with focal RT (*P* = .118; Fig. 2c). In the subgroup with PF tumors, CSI-RT2 was associated with a 5-year PFS of 50%, compared with 9% with focal RT2 (*P* = .102; Fig. 2d).

To explore why CSI was associated with an OS benefit but not with a PFS benefit, we analyzed the subset of patients who received CSI-RT2 and subsequently had recurrence after RT2 (Table 2). Three patients received further salvage therapy; all had local recurrences and were treated with additional local therapies (surgery [n = 3] for 1 patient who received third course radiation therapy). All were alive at the last follow-up and had no evidence of active disease after salvage therapy post-RT2 local recurrence, though 1 individual (patient 1) emigrated without further follow-up information. Therefore, these 3 patients were counted as PFS events post-RT2, but remained alive and without evidence of disease at the last known follow-up.

**Table 2:** Description of patients who recurred after repeat radiation therapy with craniospinal irradiation and subsequent therapies

Patient	Age/sex	Pathology	RT1	Surgery 2	RT2	Recurrence pattern	Surgery 3	Survival from RT1
1	26/F	Grade 3, ZFTA	Focal, 59.4 Gy	NTR for a distant brain recurrence	14 mo post-RT1: CSI 36 Gy, focal boost 55.8 Gy	4 mo post-RT2: local recurrence within the surgery 2 tumor bed	GTR, followed by RT3	NED at last follow-up, but with necrosis after RT3 Time to last follow-up from RT2: 9 mo
2	3/M	Grade 3, PF-A	Focal, 59.4 Gy	GTR for a local recurrence	31 mo post-RT1: CSI 23.4 Gy, focal boost 54 Gy	40 mo post-RT2: local recurrence	GTR	NED since RT2 Time to last follow-up from RT2: 100 mo
3	7/M	Grade 3, ZFTA	Focal, 59.4 Gy	GTR for a local recurrence	16 mo post-RT1: CSI 23.4 Gy, focal boost 54 Gy	44 mo post-RT2: local recurrence	GTR	NED since RT2 Time to last follow-up from RT2: 101 mo

*Abbreviations:* CSI = craniospinal irradiation; F = female; GTR = gross total resection; M = male; NED = no evidence of disease; NTR = near-total resection; PF-A = posterior fossa tumor group A; RT = radiation therapy; RT1 = initial radiation therapy; RT2 = reirradiation; RT3 = third course of radiation therapy; ZFTA = zinc finger translocation associated.

### Disseminated ependymoma

**Figure 3:**
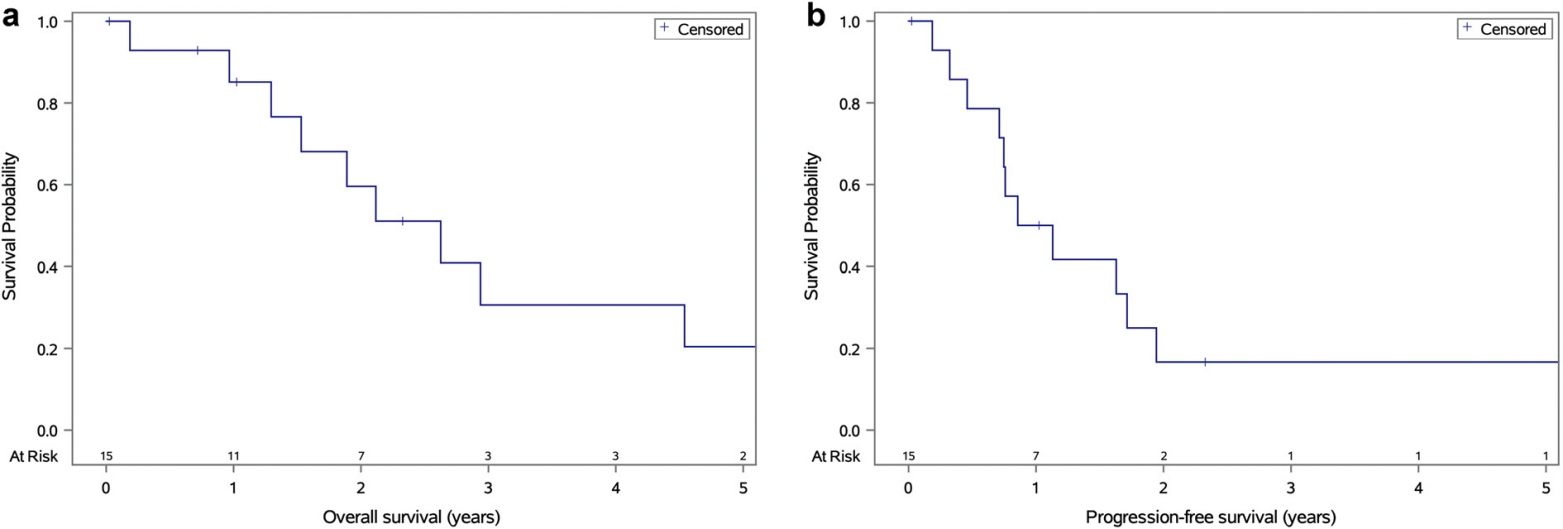
Outcomes of patients with disseminated-only or synchronous local and disseminated failures. (a) The 5-year overall survival was 20% (95% CI, 3.3%-47.6%), and (b) the 5-year progression-free survival was17% (95% CI, 2.8%-40.9%).

In our cohort, 15 patients presented with either disseminated-only or synchronous local and disseminated recurrence. Among these, 14 received CSI-RT2 and a sequential focal RT2 boost. The 5-year OS in this group was 20% (CI, 3.3%-47.6%; Fig. 3a), indicating a poor prognosis for disseminated disease. Similarly, the 5-year PFS rate was 17% (CI, 2.8%-40.9%; Fig. 3b), reflecting the challenges of achieving disease control over time.

Patterns of failure in all patients, stratified by recurrence type after RT1 and RT2 fields, are described in Table 3. Only 1 locally recurrent patient (out of 10) developed subsequent disseminated tumor recurrence when CSI was incorporated into RT2.

**Table 3:** Patterns of failure after reirradiation in patients who had local recurrence after initial radiation therapy

Patterns of failure	All patients (N = 48)		Patients with local recurrences after RT1, n = 33		Patients with disseminated recurrence after RT1, n = 15	
	Focal RT2 (n = 24)	CSI-RT2 (n = 24)	Focal RT2 (n = 23)	CSI-RT2 (n = 10)	Focal RT2 (n = 1)	CSI-RT2 (n = 14)
Local failure, n	11	6	11	4	0	2
Disseminated failure, n	7	9	6	1*	1	8
No failure, n	6	9	6	5	0	4

*Abbreviations:* CSI = craniospinal irradiation; RT1 = initial radiation therapy; RT2 = reirradiation.

⁎ This patient was treated with CSI-RT2 to 36 Gy.

### Toxicities and functional outcomes

Radionecrosis was observed in 5 patients in our cohort after RT2 (grade 3: 4 patients; grade 2: 1 patient). Of these, 2 were treated with dexamethasone alone, 2 required bevacizumab, and 1 underwent hyperbaric oxygen therapy.

Functional outcomes were assessed in 19 long-term survivors without evidence of active disease. Ten patients exhibited mild to moderate neurocognitive impairment, often necessitating individualized education plans in school. Three patients were enrolled in postsecondary education, including 1 who had previously received CSI to a dose of 36 Gy. More than half of the surviving patients (n = 11) did not develop, or had not yet developed, neuroendocrine deficits at the last follow-up. Notably, 1 patient developed a secondary malignancy, epithelioid hemangioendothelioma of the fourth ventricle, approximately 12 years after receiving CSI-RT2. It was surgically resected and treated with everolimus for systemic involvement. This patient remains free of recurrent ependymoma and is alive, well, and gainfully employed as a health care provider, more than 3 years after diagnosis of hemangioendothelioma.

## Discussion

In this retrospective, single-institution study spanning over 2 decades, we report long-term outcomes among children and adults with recurrent intracranial ependymoma treated with RT2. In this cohort of 48 patients with recurrent intracranial ependymoma, predominantly pediatric and with PF involvement, GTR of recurrent disease was significantly associated with improved OS. Among patients with local recurrence, CSI-RT2 improved 5-year OS compared with focal RT. Notably, no statistically significant PFS benefit was observed, likely due to successful salvage of subsequent local recurrences post–CSI-RT2. Patients with disseminated disease had poor outcomes despite aggressive treatment with CSI.

The results of this study highlight several important considerations in the management of intracranial ependymoma: the importance of achieving GTR of residual disease when surgically feasible, and the benefit of CSI-RT2 in patients with local recurrence.

The importance of local control in this disease cannot be overemphasized; achieving complete resection of locally recurrent ependymoma is key to maximizing treatment success. This is consistent with prior reports that RT2 and complete resection of recurrences are critical for achieving control of recurrent ependymoma.^4^^,^^11^ Our cohort highlights the importance of attaining local control, even in the setting of subsequent local recurrence after RT2. The data also reinforce the important role of CSI-RT2 in improving tumor control in patients with locally recurrent ependymoma. Thus, in our cohort with long-term follow-up, CSI-RT2 may be an important component and was associated with better OS, particularly when combined with complete resection of local recurrences. Due to the small sample size, we were unable to robustly compare outcomes among patients with supratentorial primary tumors treated with or without CSI.

Prior studies have shown that RT2 at relapse is a safe, feasible, and potentially curative treatment for intracranial ependymoma.^12^ A review by De Pietro et al^13^ summarized multiple studies, noting that CSI-RT2 was associated with improved survival and disease control. This conclusion is based in part on data from our institution, which found an association between CSI‑RT2 and improved local control in PF ependymoma, due to a reduction in distant relapse.

Our data also emphasize the importance of surgical resection. A retrospective study showed that in locally recurrent ependymomas, GTR led to a 5-year PFS of 40% versus 27% with STR; 5- and 10-year OS estimates were 58% and 50% with GTR, respectively, versus 51% and 17% with STR, respectively.^14^ A multi-institutional study of treatments at relapse found that, irrespective of whether the recurrence was localized or disseminated, treatments that included surgery and/or RT2 had better outcomes.^15^

In our cohort, patients with disseminated recurrence had uniformly poor outcomes despite the almost universal use of CSI. Future strategies need to incorporate novel systemic therapies to improve outcomes for this high-risk group.

A strength of this study is its long median follow-up (9.1 years), which enabled robust assessment of long-term survival in a disease known for late recurrences. However, this study also has limitations inherent to its retrospective design, including selection bias in treatment modality, variability in prior therapies and therapies at recurrence (particularly CSI-RT2 dose), and incomplete molecular data in a subset of patients. The small number of adults also limits generalizability to that population. We were unable to determine whether the extent of resection (GTR) and CSI were independently associated with survival; future work should expand the patient sample to permit multivariable analyses to further evaluate these 2 variables together. Our data also do not allow a robust comparison of CSI-RT2 dosing (23.4 Gy vs 36 Gy). In patients with local recurrence after RT1 who were treated with CSI-RT2, 1 patient had a distant recurrence; that individual was treated with CSI-RT2 to 36 Gy.

## Conclusions

Our findings support a treatment paradigm in which GTR is followed by CSI-RT2 for patients with locally recurrent intracranial ependymoma, which is associated with superior patient survival. These results reinforce the value of aggressive local control with surgery and regional control with CSI-RT2 to maximize disease control in children and adults with recurrent ependymoma. Additional work is needed to independently confirm the prognostic significance of both the extent of resection (GTR) and the RT field (CSI) in larger data sets.

## Disclosures

Ying Ying Sum received salary support from the Princess Margaret Cancer Foundation. Derek S. Tsang is a consultant for Need unrelated to this study.

## References

[1] Merchant T.E., Li C., Xiong X., Kun L.E., Boop F.A., Sanford R.A. (2009). Conformal radiotherapy after surgery for paediatric ependymoma: A prospective study. Lancet Oncol.

[2] Mac Donald S.M., Sethi R., Lavally B. (2013). Proton radiotherapy for pediatric central nervous system ependymoma: Clinical outcomes for 70 patients. Neuro Oncol.

[3] Merchant T.E., Bendel A.E., Sabin N.D. (2019). Conformal radiation therapy for pediatric ependymoma, chemotherapy for incompletely resected ependymoma, and observation for completely resected, supratentorial ependymoma. J Clin Oncol.

[4] Mak D.Y., Laperriere N., Ramaswamy V. (2021). Reevaluating surgery and re-irradiation for locally recurrent pediatric ependymoma-A multi-institutional study. Neurooncol Adv.

[5] Ramaswamy V., Hielscher T., Mack S.C. (2016). Therapeutic impact of cytoreductive surgery and irradiation of posterior fossa ependymoma in the molecular era: A retrospective multicohort analysis. J Clin Oncol.

[6] Liu A.K., Foreman N.K., Gaspar L.E., Trinidad E., Handler M.H. (2009). Maximally safe resection followed by hypofractionated re-irradiation for locally recurrent ependymoma in children. Pediatr Blood Cancer.

[7] Zacharoulis S., Ashley S., Moreno L., Gentet J.C., Massimino M., Frappaz D. (2010). Treatment and outcome of children with relapsed ependymoma: A multi-institutional retrospective analysis. Childs Nerv Syst.

[8] Messahel B., Ashley S., Saran F., Children's Cancer Leukaemia Group Brain Tumour Committee (2009). Relapsed intracranial ependymoma in children in the UK: Patterns of relapse, survival and therapeutic outcome. Eur J Cancer.

[9] Bouffet E., Hawkins C.E., Ballourah W. (2012). Survival benefit for pediatric patients with recurrent ependymoma treated with reirradiation. Int J Radiat Oncol Biol Phys.

[10] Tsang D.S., Murray L., Ramaswamy V. (2019). Craniospinal irradiation as part of re-irradiation for children with recurrent intracranial ependymoma. Neuro Oncol.

[11] Liu Z.M., Han Z., Wang J.M. (2022). Treatment and outcome of pediatric intracranial ependymoma after first relapse. J Neurooncol.

[12] Lobón M.J., Bautista F., Riet F. (2016). Re-irradiation of recurrent pediatric ependymoma: Modalities and outcomes: A twenty-year survey. Springerplus.

[13] De Pietro R., Zaccaro L., Marampon F., Tini P., De Felice F., Minniti G. (2023). The evolving role of reirradiation in the management of recurrent brain tumors. J Neurooncol.

[14] Aldave G., Okcu M.F., Ruggieri L. (2023). The role of surgery in recurrent ependymomas. J Neurosurg Pediatr.

[15] Desrousseaux J., Claude L., Chaltiel L. (2023). Respective roles of surgery, chemotherapy, and radiation therapy for recurrent pediatric and adolescent ependymoma: A national multicentric study. Int J Radiat Oncol Biol Phys.

